# Time to Evaluate the Clinical Repercussions of Zika Virus Vertical Transmission? A Systematic Review

**DOI:** 10.3389/fpsyt.2021.699115

**Published:** 2021-08-30

**Authors:** Yasmin Notarbartolo di Villarosa do Amaral, Jocieli Malacarne, Paloma Glauca Brandão, Patrícia Brasil, Karin Nielsen-Saines, Maria Elisabeth Lopes Moreira

**Affiliations:** ^1^Department of Pediatrics, Instituto Fernandes Figueira, Rio de Janeiro, Brazil; ^2^Department of Acute Febrile Illnesses, Instituto Nacional de Infectologia, Rio de Janeiro, Brazil; ^3^Department of Pediatrics, University of California, Los Angeles, Los Angeles, CA, United States

**Keywords:** zika virus, systematic review, neurological repercussion, clinical repercussion, neurodevelopment

## Abstract

**Background:** Vertical transmission of Zika Virus (ZIKV) can be associated with several clinical features in newborn infants. The goal of the present review was to analyze the current state of knowledge regarding clinical repercussions following perinatal exposure to ZIKV in children up to 3 years of age.

**Methods:** A systematic review of published studies was carried out, without the restriction of language or date of publication, identified in the databases PubMed, Virtual Health Library (BVS), Scopus, and Web of Science and the catalog for CAPES theses and dissertations. According to the proposed flowchart, the bibliographic search resulted in 1,563 papers. Of these, according to the eligibility criteria, 70 were selected for systematic review; all were published between 2016 and 2021.

**Results:** Regarding clinical findings, 19 papers evaluated clinical imaging alterations, 21 ophthalmic manifestations, and 39 evaluated the central nervous system; of these, 15 analyzed neuro-psychomotor development. The remainder evaluated audiological (*n* = 14), nutritional (*n* = 14), orthopedic (*n* = 7), cardiorespiratory (*n* = 5), genitourinary (*n* = 3) or endocrinological (*n* = 1) manifestations.

**Conclusion:** It is critical for studies to continue monitoring children with antenatal ZIKV exposure as they grow, given the unknown long-term repercussions of ZIKV and the recognized postnatal complications of this infection during pregnancy. Broader descriptions of observed clinical findings are also important in order to characterize the entire spectrum of disease in children.

**Systematic Review Registration:** PROSPERO REGISTER: CRD42020205947.

## Background

Zika virus (ZIKV) was first reported in East Africa in the 1950 s. In 2007, global attention emerged following an outbreak in Micronesia, and in the following decade, on the island of Yap, in the French Polynesia. The virus spread widely in other Pacific islands over the years, before emerging as a widespread epidemic throughout Latin America (1, 2). In 2015, with the arrival of ZIKV in Brazil, the first studies reported descriptions of women with fever and rash during pregnancy and a possible relationship with congenital microcephaly (3–5). The hypothetical relationship between ZIKV infection in pregnancy and subsequent abnormal newborn findings arose after a very large increase in microcephaly cases was observed in Brazil a few months after ZIKV circulation was identified in the country. Due to its catastrophic repercussions to newbor infants, the World Health Organization (WHO) declared Zika virus a Public Health Emergency in 2016. Gradually over 2017, ZIKV cases declined consistently across the world, although certain tropical areas of the globe became endemic for ZIKV infection, including Central and South America, the Caribbean, and southern Asia. Outbreaks were reported in 2018 in India and Angola, and in France, a locally acquired infection was reported in 2019. One of the driving forces behind the rapid ZIKV epidemic spread was global warming and population mobility which greatly contributed to an increase in the environmental span of *Aedes sp*. mosquitoes. The possibility of new outbreaks lingers, particularly since arboviral outbreaks are notoriously cyclical. In addition, ZIKV, unlike other arboviral infections, can be transmitted by sexual contact. Therefore, pregnant women may be infected by partners who traveled to endemic areas. Therefore, travel histories should include not only the pregnant patient but their partners as well. Since the virus can persist for extended periods of time in semen, pregnant women could be at risk for infection weeks to months following partner travel to endemic areas.

The fact that ZIKV has a very similar genomic structure to dengue viruses 1–4, has important diagnostic implications. Arboviral flaviviruses in the same family as ZIKV include yellow fever, Japanese encephalitis, and West Nile viruses. Hepatitis C virus, another flavivirus, also shares some genomic similarities with ZIKV, which carries potential antiviral treatment implications. Over time ZIKV evolved from the African lineage to the Asian lineage (there is 90% homology between strains), and potentially acquired higher teratogenic potential during the process. The Asian strain of ZIKV was responsible for the recent pandemic.

Although ZIKV infection is generally asymptomatic, 20% of patients develop mild symptoms. The clinical features resemble that of rubella virus infection. If symptoms occur, they are present 7–10 days following exposure. Most prominent findings are a maculopapular pruritic rash, arthralgia and conjunctival erythema. Fever is rare and, if present, low grade. Rash, pruritus, conjunctival hyperemia, no fever, no petechiae and no anorexia are used as a ZIKV case definition in endemic settings, where dengue and chikungunya are also prevalent. ZIKV infection is typically self-limited with resolution of symptoms within 1 week. Most patients recover without complications, including pregnant women.The absence of clinical symptoms of ZIKV during pregnancy, however, does not indicate no risk of clinical repercussions to infants. Women with asymptomatic disease can deliver infants with microcephaly. Virus load during maternal infection, disease severity and frequency of symptoms, as well as prior dengue immunity have not been predictive of infant outcomes at birth.

The Centers for Disease Control and Prevention (CDC) coined the term Congenital Zika Syndrome (CZS) which refers to infants most severely affected by antenatal ZIKV exposure. Nevertheless, many studies demonstrated a spectrum of clinical manifestations in children ranging from absent findings to severe microcephaly. CZS is defined as a constellation of findings at birth including: (1) severe microcephaly (>3 SD below the mean for gestational age and gender); (2) brain abnormalities (subcortical calcifications, ventriculomegaly, cortical thinning, gyral pattern anomalies, hypoplasia of the cerebellum, or corpus callosum anomalies); (3) ocular findings; (4) congenital contractures, also known as arthrogryposis; and (5) neurologic impairment. Microcephaly rates range from 3 to 7% in prospective studies. Most common abnormalities include cerebral calcifications, cortical developmental malformations (lissencephaly, pachygyria, agyria), ventriculomegaly due to brain atrophy, posterior fossa alterations including brainstem or cerebellar hypoplasia, corpus callosum abnormalities, enlarged extra-axial cerebrospinal fluid spaces, and enlarged cisterna magna. Ophthalmologic and sensorineural hearing loss have been reported in 7 and 12% of infants, respectively, followed since the time of maternal infection. They prevail in children with other CNS findings but can also be an isolated finding. Eye manifestations include abnormalities of the retinal pigment epithelium of the macula, optic nerve hypoplasia, chorioretinal atrophy; other abnormalities are colobomas and microphthalmia. Abnormal visual function is identifiable in early infancy among affected children. Eye abnormalities do not tend to progress. Another interesting observation, which highlights some similarities with congenital rubella syndrome is that 10% of children with *in utero* ZIKV exposure had congenital heart defects in prospective studies. Longer term outcome studies demonstrated that 15% of children may have severe neurodevelopmental problems and sensorineural abnormalities by 3 years of age. Conversely, not all children with abnormalities at birth have later neurodevelopmental repercussions. In the same way, infants found to be normal at birth following maternal infection during pregnancy might have abnormal developmental outcomes years later. Studies demonstrated that close to 1/3 of infants with antenatal ZIKV exposure have below average neurodevelopment or abnormal eye or hearing findings, Secondary microcephaly, which is microcephaly occurring after birth, as well as a higher rate of ASD have been noted in children exposed to antenatal ZIKV, underscoring that long term follow-up is necessary.

ZIKV has been shown to cross the placenta and infect placental macrophages. This disrupts neural progenitor cell evolution, leading to microcephaly in animal models. Maternal infection earlier in pregnancy leads to more severe fetal outcomes. CNS malformations are more common with first and second trimesters infections. Late term fetal demise can occur due to placental vascular involvement with focal necrotic vasculitis and placental failure. In summary, adverse outcomes due to ZIKV infection have been described across all trimesters of pregnancy. Miscarriages and fetal growth restriction have also been described. The virus can induce CNS calcifications and bone fusion; craniosynostosis may be present in congenital ZIKV infection.

Congenital ZIKV infection has become widely recognized since its original description. Microcephaly is defined as a head circumference of <2 or more standard deviations from the benchmark for gender, age, or gestational age, per the Brazilian Ministry of Health (6). The spectrum of congenital disabilities linked to ZIKV besides microcephaly, such as eye alterations, craniofacial disproportion, and joint and limb deformities, characterize Congenital ZIKV Syndrome (CZS) (7). As previously discussed, clinical alterations and subsequent developmental delays are widely described in babies born without microcephaly, in some cases infants with no stigmata of CZS (8–12). However, there is very little information about future clinical implications of antenatal ZIKV infection in the long term, and this is the target of several studies.

## Methods

A systematic review was undertaken to analyze the current state of knowledge regarding repercussions of vertical exposure to ZIKV on child health. The search for pertinent studies was carried out using databases of the Virtual Health Library (BVS), MEDLINE via PubMed, Web of Science, and Scopus via Capes journals portal, CAPES thesis, and dissertation catalogs.

**Figure 1:**
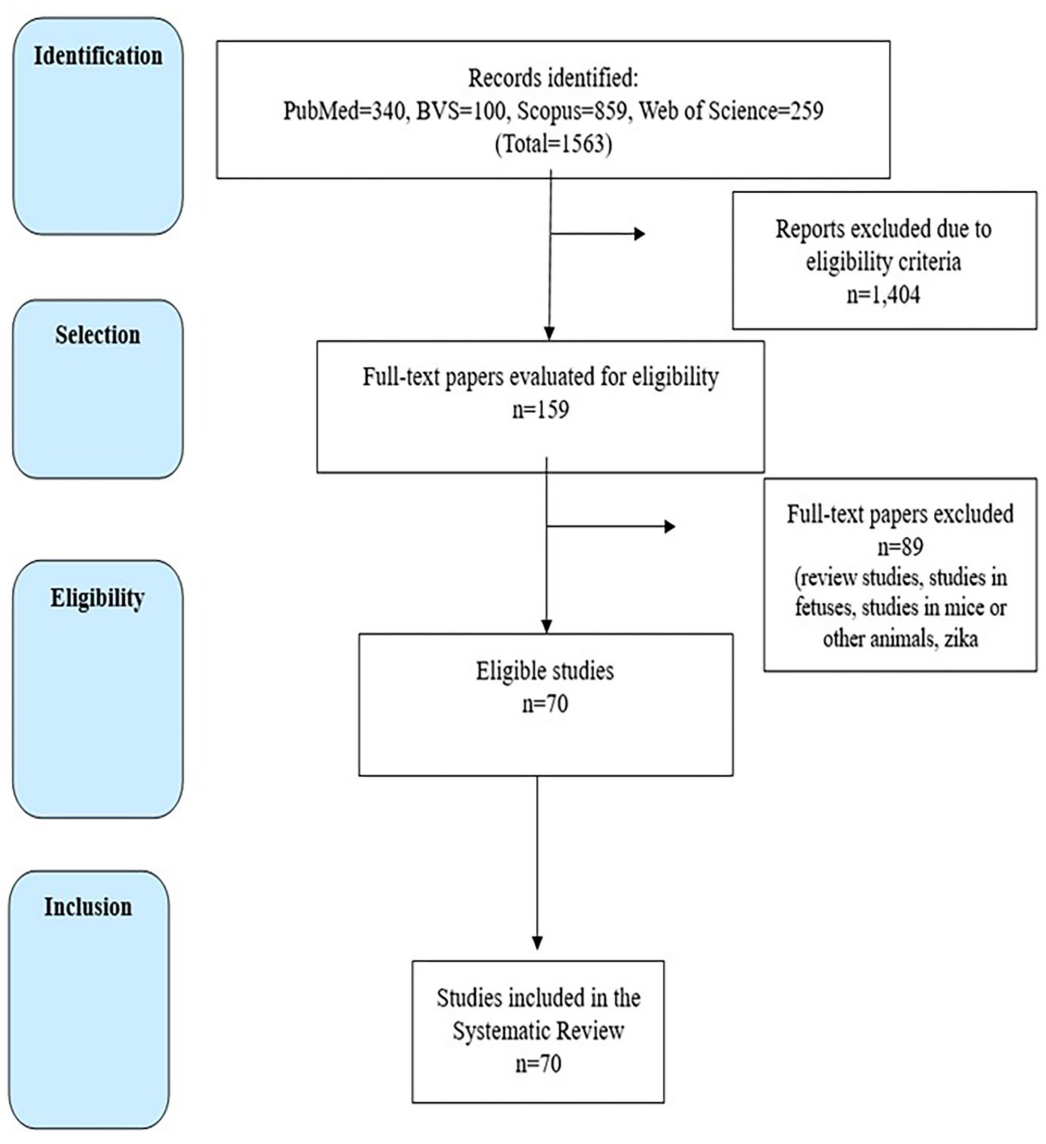
Inclusion flowchart (PRISMA).

This comprehensive review was undertaken to address the following question: “*What is the impact of vertical exposure to ZIKV on clinical, nutritional, and neurodevelopmental aspects in children up to 3 years of age?*” This question was formulated per the PICO acronym. The description of this systematic review was based on the Preferred Reporting Items for Systematic Reviews (PRISMA) guidelines (13). Thus, the following steps were developed: identification of the research question, literature search, data evaluation, analysis of results, and presentation of the review (Figure 1).

The following descriptors were used for the search strategy: “Zika Virus,” “Zika Virus Infection” as search terms, along with “Zika Virus Infection/complications” or the specific clinical outcome designations: “neurogenic bladder,” “urinary bladder,” “Nutritional status,” “nutrition,” “Anthropometry,” “Hearing,” “Orthopedics,” “arthrogryposis,” “vision,” “Neurologic disease,” “Neurologic Manifestations,” “Gastrointestinal Diseases,” “Cardiovascular disease,” “Cardiovascular Abnormalities,” “neurodevelopment.” Boolean operators AND, OR, and NOT were used to relate the blocks to each other, to add at least one word from each block. This systematic review was registered and approved by the PROSPERO systematic review protocol registry database under registration number CRD42020205947.

Two independent researchers carried out the search process, which ended in January 2021, with no limits for the period of publication or language restrictions. The bibliographic search resulted in 1,563 papers. Of these, 159 were selected for full-text reading because they evaluated clinical manifestations in cohorts of children with antenatal ZIKV exposure. After extensive selection, 89 papers were excluded because they addressed topics that were not relevant to the present work, leaving 70 studies eligible for this paper, as seen in Figure 1. Eligibility criteria for manuscript selection included full text studies that reported clinical findings/outcomes in cohorts of children with documented antenatal ZIKV exposure. As such, incomplete manuscripts/abstracts, review papers, studies in fetuses, animal studies, *in vitro* studies, studies in adults only, and qualitative studies were excluded. In addition, manuscripts referenced in papers selected for this study were also investigated, however no further papers were identified. The selected publications were compared in regards to the following parameters: year of publication, study location, sample size, mean age of participants, design type, eligibility criteria, exposure period, presence of a control group, symptoms, controlled confounders in the analysis, study limitations, and main results.

## Results

**Figure 2:**
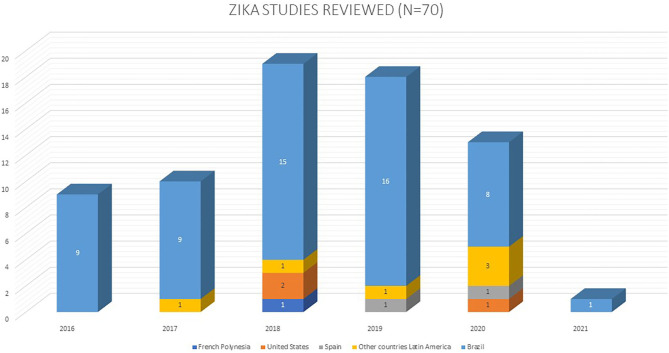
Zika studies reviewed by year of publication and country of origin.

Seventy papers published from 2016 to 2021 were selected following the search. Of these, most were conducted in Brazil (*n* = 58), followed by Colombia (*n* = 4), the U.S. (*n* = 3), Spain (*n* = 2), French Guiana (*n* = 1), Mexico (*n* = 1), and the French Polynesia (*n* = 1) (Figure 2). The sample size ranged from 1 to 5,673 participants. The population studied ranged in age from 0 days to 48 months, with 9 studies not reporting the participant age range (Table 1).

**Table 1:** Year of publication, origin, sample size and age of participants of selected studies, 2016–2021.

**References**	**Country**	**Sample size (*n*)**	**Age of follow-up**
Almeida et al. (14)	Brazil	100	Not provided
Alves et al. (15)	Brazil	24	19.9 (18– 24 months)
Aragao et al. (16)	Brazil	12	135 days
Bertolli et al. (17)	Brazil	120	24 months
Brasil et al. (5)	Brazil	207	Birth
Carvalho et al. (18)	Brazil	82	13, 2 months
Carvalho et al. (19)	Brazil	37	2, 6 (1–5 months)
Carvalho-Sauer et al. (20)	Brazil	393	Birth
Contreras-Capetillo et al. (21)	Mexico	3	Newborns
Costa Monteiro et al. (22)	Brazil	69	13 months
Costa Monteiro et al. (23)	Brazil	22	9 months
Cranston et al. (24)	Brazil	296	0 −48 months
de Fatima Vasco Aragao et al. (25)	Brazil	23	1 month
de Paula Freitas et al. (26)	Brazil	29	1–6 months
de Paula Guimarães et al. (27)	Brazil	69	Not provided
dos Santos et al. (28)	Brazil	21	Not provided
dos Santos et al. (29)	Brazil	65	15 months
Fandiño-Cárdenas et al. (30)	Colombia	66	Exposed: 3.5 months; Control: 3 months
Felix et al. (31)	French Guiana	2	2–4 months
Ferreira et al. (32)	Brazil	34	21 months
França et al. (33)	Brazil	24	20.5 months
Jucá et al. (34)	Brazil	115	Not provided
Kanda et al. (35)	Brazil	23	8.3 months
C Lage et al. (36)	Brazil	102	4.1 months
Leal et al. (37)	Brazil	70	0–10 months
Leal et al. (38)	Brazil	9	4 (2–7 months)
Leal et al. (39)	Brazil	1	Birth−1 month
Leal et al. (40)	Brazil	57	22.9 months
Leite et al. (41)	Brazil	45	10 months
Linden et al. (42)	Brazil	3	7–19 months
Marques Abramov et al. (43)	Brazil	19	Not provided
Melo et al. (44)	Brazil	59	14.7 months
Meneses et al. (45)	Brazil	87	Birth
Lopes Moreira et al. (10)	Brazil	104	2–18 months
Moura da Silva et al. (46)	Brazil	48	1–8 months
Mulkey et al. (47)	Colombia	70	Birth−18 months
Nielsen-Saines et al. (11)	Brazil	216	18 months
Oliveira-Filho et al. (48)	Brazil	27	101 days
Orofino et al. (49)	Brazil	186	97 (1–376 days)
Ospina et al. (50)	Colombia	5,673	Birth
Pacheco et al. (51)	Colombia	60	20–30 months
Peçanha et al. (52)	Brazil	84	1st Moment: 9.7 months 2nd Moment: 15.3 months
Petribu et al. (53)	Brazil	37	1st Moment: 1 to 138 days (median of 11.5 days) 2nd Moment: 105 to 509 days (median of 415 days)
Pinato et al. (54)	Brazil	136	5–24 months
Pone (55)	Brazil	106	Not provided
Pool et al. (56)	Brazil	110	Newborn period
Rajapakse et al. (57)	United States	4	3–10 days of life; 1–86 days
Rice et al. (58)	United States	1,450	≥12 months
Rocha et al. (59)	Brazil	174	9 months
Roma et al. (60)	Brazil	20	Newborns
Santana et al. (61)	Brazil	18	21.5 months
Satterfield-Nash et al. (62)	Brazil	19	22 months
Soares et al. (63)	Brazil	115	Birth – 3 months
Soriano-Arandes et al. (64)	Spain	143	1, 4, 9, 12, 18, and 24 months
Subissi et al. (65)	French Polynesia	123	23 months
Sulleiro et al. (66)	Spain	1	24 months
Trigueiro et al. (67)	Brazil	20	Not provided
Tsui et al. (68)	Brazil	224	44 days (12–99 days)
van der Linden et al. (69)	Brazil	21	16–30 months (mean 16 months at the time of the last examination)
van der Linden et al. (70)	Brazil	13	05–12 months
van der Linden et al. (71)	Brazil	7	Not provided
de Vasconcelos et al. (72)	Brazil	22	36 months
Ventura et al. (73)	Brazil	40	2.2 months
Ventura et al. (73)	Brazil	32	5.7 (4–7 months)
Ventura et al. (74)	Brazil	204	Exposed: 8.5 months (6–13 months) Controls: 8.4 months (5–12 months)
Veras Gonçalves et al. (75)	Brazil	30	41 months
Verçosa et al. (76)	Brazil	70	3 months
Walker et al. (77)	United States	95	Newborn period
Zin et al. (12)	Brazil	112	Not provided
Zin et al. (78)	Brazil	173	3–6 months

**Figure 3:**
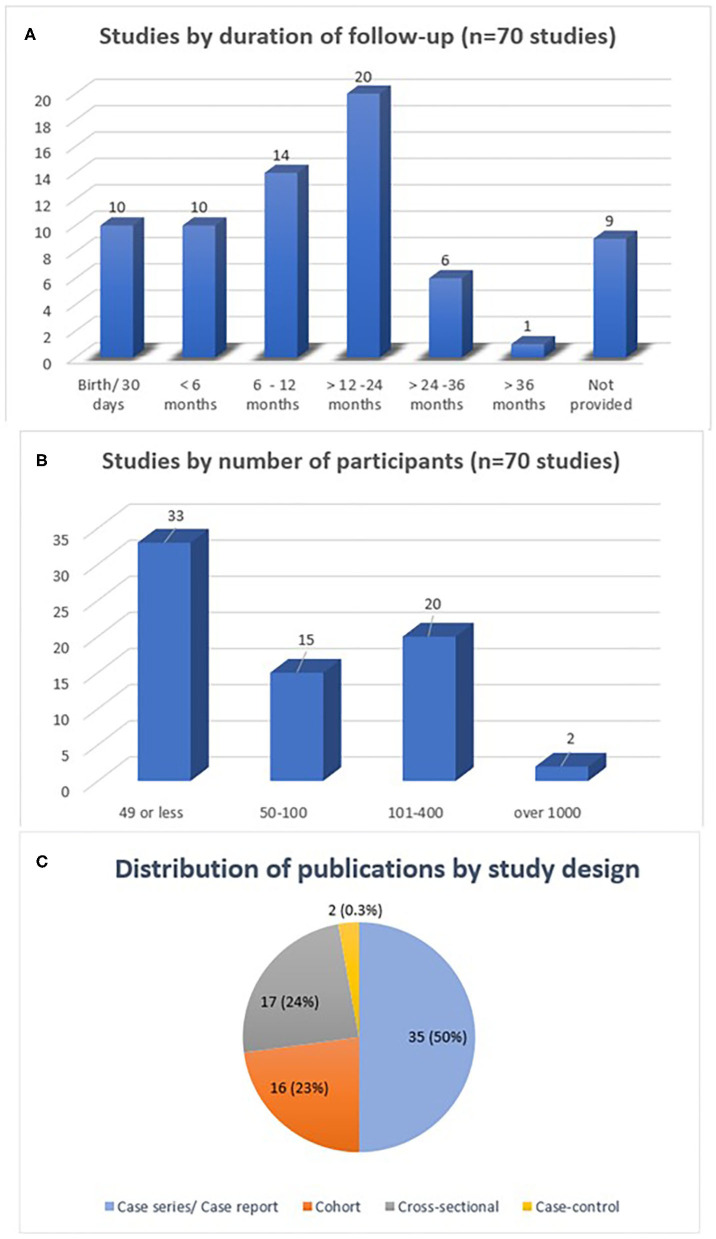
Description of studies reviewed by duration of follow-up **(A)**, number of participants **(B)** and study design **(C)**.

Most studies were (*n* = 37) descriptive in design, such as case series or case reports, followed by cross-sectional studies (*n* = 17), cohort studies (*n* = 14), and case-control studies (*n* = 2). Information on study limitations was described in 40 studies; the most prevalent limitation was the limited sample size, lack of a control group, type or lack of laboratory confirmation for ZIKV, loss to follow-up, and use of secondary data for analysis. Details on duration of follow-up, sample size and study design are shown in Figure 3.

Concerning clinical findings, 19 papers evaluated clinical imaging alterations, 21 ophthalmic manifestations, 39 the central nervous system, including 15 which evaluated neuro-psychomotor development. Additional studies included audiological (*n* = 14), nutritional (*n* = 14), orthopedic (*n* = 7), cardiorespiratory (*n* = 5), genitourinary (*n* = 3) or endocrinological (*n* = 1) manifestations. It is noteworthy that some articles described more than one organ system and multiple clinical findings (Table 2).

**Table 2:** Study design, clinical abnormalities and development screening test, 2016–2021.

**References**	**Study design**	**Clinical abnormalities**	**Developmental screening test**
Almeida et al. (14)	Case series	Hearing abnormalities; Eye abnormalities	–
Alves et al. (15)	Case series	Neurodevelopmental delays	Denver Development Screening Test II
Aragao et al. (16)	Case series	Joint and limb deformities	–
Bertolli et al. (17)	Cohort	Neurodevelopmental delays	ASQ *3*
Brasil et al. (5)	Cohort	Cerebral abnormalities (Microcephaly, Calcification, Hydrocephaly, Cerebral atrophy, Cerebellar alterations)	–
Carvalho et al. (18)	Case series	Neurological abnormalities; Neurodevelopmental delays; Cerebral abnormalities (Microcephaly, Calcification, Hydrocephaly, Cerebral atrophy, Cerebellar alterations)	–
Carvalho et al. (19)	Case series	Neurological abnormalities	–
Carvalho-Sauer et al. (20)	Cross-sectional	Growth and nutrition	–
Contreras-Capetillo et al. (21)	Case series	Joint and limb deformities; Growth and nutrition; Cerebral abnormalities (Microcephaly, Calcification, Hydrocephaly, Cerebral atrophy, Cerebellar alterations)	–
Costa Monteiro et al. (22)	Case series	Genito-urinary abnormalities (Criptorquidia, Neurogenic bladder)	–
Costa Monteiro et al. (23)	Case series	Genito-urinary abnormalities (Criptorquidia, Neurogenic bladder)	–
Cranston et al. (24)	Cohort	Cardiological abnormalities; Hearing abnormalities; Eye abnormalities; Neurological abnormalities; Growth and nutrition; Neurodevelopmental delays	Bayley III
de Fatima Vasco Aragao et al. (25)	Retrospective case series	Cerebral abnormalities (Microcephaly, Calcification, Hydrocephaly, Cerebral atrophy, Cerebellar alterations)	–
de Paula Freitas et al. (26)	Case series	Eye abnormalities	–
de Paula Guimarães et al. (27)	Case series	Hearing abnormalities; Eye abnormalities; Neurological abnormalities	–
dos Santos et al. (28)	Descriptive Longitudinal Study	Growth and nutrition	–
dos Santos et al. (29)	Descriptive Longitudinal Study	Growth and nutrition	–
Fandiño-Cárdenas et al. (30)	Cohort	Hearing abnormalities	–
Felix et al. (31)	Case report	Neurological abnormalities	–
Ferreira et al. (32)	Cross-sectional	Neurodevelopmental delays	Common Brief ICF Core Set for CP
França et al. (33)	Cross-sectional	Growth and nutrition; Neurodevelopmental delays	Bayley III
Jucá et al. (34)	Case series	Cerebral abnormalities (Microcephaly, Calcification, Hydrocephaly, Cerebral atrophy, Cerebellar alterations)	–
Kanda et al. (35)	Cross-sectional	Neurological abnormalities	–
C Lage et al. (36)	Cross-sectional	Joint and limb deformities; Hearing abnormalities; Eye abnormalities; Neurological abnormalities	–
Leal et al. (37)	Case series	Hearing abnormalities	–
Leal et al. (38)	Case series	Gastrointestinal/pulmonary abnormalities	–
Leal et al. (39)	Case report	Hearing abnormalities	–
Leal et al. (40)	Cross-sectional study nested in a cohort	Adenoid hypertroph	–
Leite et al. (41)	Cross-sectional	Hearing abnormalities	–
Linden et al. (42)	Case series	Joint and limb deformities; Neurological abnormalities	–
Marques Abramov et al. (43)	Cross-sectional	Hearing abnormalities	–
Melo et al. (44)	Cross-sectional	Neurodevelopmental delays	Not provided
Meneses et al. (45)	Case series	Eye abnormalities; Neurological abnormalities; Cerebral abnormalities (Microcephaly, Calcification, Hydrocephaly, Cerebral atrophy, Cerebellar alterations); Gastrointestinal/pulmonary abnormalities	–
Lopes Moreira et al. (10)	Cohort	Neurodevelopmental delays	Bayley III
Moura da Silva et al. (46)	Case series	Gastrointestinal/pulmonary abnormalities; Joint and limb deformities; Neurological abnormalities; Cerebral abnormalities (Microcephaly, Calcification, Hydrocephaly, Cerebral atrophy, Cerebellar alterations)	–
Mulkey et al. (47)	Cohort	Neurodevelopmental delays	Warner Initial Developmental Evaluation of Adaptive and Functio-l Skills (WIDEA) and the Alberta Infant Motor Scale (AIMS)
Nielsen-Saines et al. (11)	Cohort	Hearing abnormalities; Neurodevelopmental delays	Bayley III
Oliveira-Filho et al. (48)	Cohort	Gastrointestinal/pulmonary abnormalities; Neurological abnormalities; Growth and nutrition; Cerebral abnormalities (Microcephaly, Calcification, Hydrocephaly, Cerebral atrophy, Cerebellar alterations)	–
Orofino et al. (49)	Cross-sectional	Cardiological abnormalities	–
Ospina et al. (50)	Retrospective cohort	Eye abnormalities; Growth and nutrition; Cerebral abnormalities (Microcephaly, Calcification, Hydrocephaly, Cerebral atrophy, Cerebellar alterations)	–
Pacheco et al. (51)	Descriptive study	Hearing abnormalities; Eye abnormalities; Neurological abnormalities	–
Peçanha et al. (52)	Case series	Neurodevelopmental delays	Bayley III
Petribu et al. (53)	Case series	Cerebral abnormalities (Microcephaly, Calcification, Hydrocephaly, Cerebral atrophy, Cerebellar alterations)	–
Pinato et al. (54)	Cross-sectional	Neurological abnormalities	–
Pone (55)	Cross-sectional	Cerebral abnormalities (Microcephaly, Calcification, Hydrocephaly, Cerebral atrophy, Cerebellar alterations)	–
Pool et al. (56)	Retrospective cohort	Hearing abnormalities; Eye abnormalities; Neurological abnormalities; Cerebral abnormalities (Microcephaly, Calcification, Hydrocephaly, Cerebral atrophy, Cerebellar alterations)	–
Rajapakse et al. (57)	Case series	Joint and limb deformities; Gastrointestinal/pulmonary abnormalities	–
Rice et al. (58)	Descriptive study	Neurodevelopmental delays	Not provided
Rocha et al. (59)	Case-control	Cerebral abnormalities (Microcephaly, Calcification, Hydrocephaly, Cerebral atrophy, Cerebellar alterations)	–
Roma et al. (60)	Case series	Hearing abnormalities; Eye abnormalities; Cerebral abnormalities (Microcephaly, Calcification, Hydrocephaly, Cerebral atrophy, Cerebellar alterations)	–
Santana et al. (61)	Case series	Gastrointestinal/pulmonary abnormalities; Cardiological abnormalities; Eye abnormalities; Neurological abnormalities; Neurodevelopmental delays; Cerebral abnormalities (Microcephaly, Calcification, Hydrocephaly, Cerebral atrophy, Cerebellar alterations)	Not provided
Satterfield-Nash et al. (62)	Case series	Hearing abnormalities; Neurological abnormalities; Neurodevelopmental delays	ASQ 3
Soares et al. (63)	Cohort	Growth and nutrition	–
Soriano-Arandes et al. (64)	Cohort	Hearing abnormalities; Neurological abnormalities	–
Subissi et al. (65)	Case-control	Neurodevelopmental delays	Not provided
Sulleiro et al. (66)	Case report	Neurological abnormalities; Growth and nutrition	–
Trigueiro et al. (67)	Cross-sectional	Eye abnormalities	–
Tsui et al. (68)	Case series	Eye abnormalities	–
van der Linden et al. (69)	Descriptive study	Neurological abnormalities	–
van der Linden et al. (70)	Case series	Neurological abnormalities; Cerebral abnormalities (Microcephaly, Calcification, Hydrocephaly, Cerebral atrophy, Cerebellar alterations)	–
van der Linden et al. (71)	Cohort	Neurological abnormalities	–
de Vasconcelos et al. (72)	Case series	Genito-urinary abnormalities (Cryptorchidism/Neurogenic bladder)	–
Ventura et al. (73)	Cross-sectional	Eye abnormalities	–
Ventura et al. (73)	Cross-sectional	Eye abnormalities	–
Ventura et al. (74)	Cross-sectional	Eye abnormalities; Neurological abnormalities	–
Veras Gonçalves et al. (75)	Case series	Eye abnormalities; Endocrine disfunction	–
Verçosa et al. (76)	Case series	Eye abnormalities	–
Walker et al. (77)	Retrospective cohort	Eye abnormalities; Neurological abnormalities; Cerebral abnormalities (Microcephaly, Calcification, Hydrocephaly, Cerebral atrophy, Cerebellar alterations)	–
Zin et al. (12)	Case series	Joint and limb deformities; Eye abnormalities; Neurological abnormalities	–
Zin et al. (78)	Cross-sectional	Hearing abnormalities	–

The most prevalent clinical imaging abnormalities of the central nervous system included microcephaly, ventriculomegaly, cortical malformations, *mega cisterna magna*, hydrocephalus, cerebellum or brain stem hypoplasia, and cerebral calcifications, especially at the junction between the cortical and subcortical white matter (16, 18, 21, 25, 34, 45, 46, 48, 50, 53, 56, 59–61, 75, 77). One manuscript also reported a decline in head circumference growth (70). Regarding abdominal imaging, there were no characteristic abnormalities identified in ZIKV exposed children that differed from descriptions in the general population. Imaging results were normal in 95.3% of 106 children who underwent abdominal ultrasound (55). Of five patients with abnormal abdominal ultrasounds, one (16.6%) had a splenic cyst, one (16.6%) had a diaphragmatic eventration, one (16.6%) had biliary lithiasis, one (16.6%) had multi-cystic dysplastic kidney, and two (33.4%) had a dilated renal pelvis. The prevalence of these alterations was 1.9% for renal pelvis dilatation and 0.9% for other abnormalities (55).

All the papers that evaluated the central nervous system found neurological alterations, and the main ones were seizure, epilepsy, irritability, pyramidal syndrome, sleep disorders, and hyperexcitability (5, 12, 18, 19, 27, 31, 35, 36, 42, 45, 46, 48, 50, 51, 54, 56, 61, 62, 66, 69–71, 74, 77). Regarding neuro-psychomotor development, all 15 papers reported motor, cognitive, or language delay (10, 11, 15, 17, 18, 24, 32, 33, 44, 47, 52, 58, 61, 62, 65). Noteworthy is that, in one paper, the authors reported autism spectrum disorder in three previously healthy children in the second year of life (11).

All studies that performed ophthalmological evaluations exposed some alteration, such as microphthalmia, fundoscopic alterations, macular atrophy, optic nerve abnormalities, strabismus and visual acuity defects (12, 14, 27, 36, 45, 50, 51, 56, 60, 61, 67, 68, 73–79). Twelve of 14 papers that evaluated audiological manifestations in children reported hearing disorders (11, 14, 27, 36, 37, 39, 43, 51, 56, 60, 62, 64) and two did not observe any abnormalities (30, 41).

Two papers reported unilateral diaphragmatic paralysis (45, 57), and another two found echocardiographic abnormalities (49, 61). These abnormalities were characterized by dilatation of the right atrium and the right ventricle, demonstrating an overload of the right heart chambers. In a study of children with Zika-related microcephaly, adenoid hypertrophy and symptoms of respiratory obstruction were reported (40).

Regarding genitourinary characteristics, studies reported neurogenic bladder and cryptorchidism (22, 23, 72). The most common orthopedic alteration was arthrogryposis (12, 16, 21, 36, 42, 46, 57). All papers that evaluated gastrointestinal manifestations reported dysphagia (38, 46, 48, 61). Regarding the nutritional status of children exposed to antenatal ZIKV, nine papers found anthropometric alterations such as low birth weight and growth retardation (14, 20, 21, 28, 29, 33, 48, 50, 63, 66) and one study observed endocrine dysfunctions in children with Zika-related microcephaly (75).

## Discussion

In this section we discuss the main results of manuscripts selected for this systematic review to assess the main potential clinical alterations described in antenatally ZIKV-exposed children to date.

### Neurologic, Neuroimaging and Neurodevelopmental Findings

Concerning clinical imaging alterations, (16, 34, 53, 59), severe brain damage was reported in CNS imaging studies in most children with antenatal exposure to ZIKV. The most common features identified were brain calcifications at the junction between cortical and subcortical white matter; these were associated with malformations of cortical development, usually with a simplified gyrus pattern and a predominance of pachygyria or polymicrogyria in the frontal lobes. Studies also identified an increased/dilated cisterna magna, corpus callosum abnormalities (which could be either hypoplasia or hypogenesis), ventriculomegaly, delayed myelination, and hypoplasia of the brain stem and/or cerebellum.

Petribu et al. (53) observed an interesting finding in that brain calcifications in children with confirmed or presumed CZS tended to decrease over time. This implies that brain calcifications should not be considered essential for diagnosis of CZS in children who present late to medical attention. Decrease in brain calcifications over time, however, was not associated with clinical improvement.

Santana et al. (61) reported that all children in their cohort had microcephaly, spasticity, and delayed neurological development. Epilepsy was found in 15 of 18 cases (83%). In a case series, Van Der Linden et al. (70) observed dystonic postures and other frequent and potentially disabling extrapyramidal signs. The study emphasized that early identification of extrapyramidal findings may help recognize neurodevelopmental problems and assist with implementation of rehabilitation, potentially influencing better strategies for rehabilitative interventions.

When analyzing sleep disorders in their cross-sectional study, Pinato et al. (54) showed that the CZS group of children had a shorter total sleep time and night sleep duration than the control group. However, no correlation was found between age and sleep patterns.

In a series of cases that assessed infants exposed to congenital ZIKV who were asymptomatic at birth, neurodevelopmental delay was identified through the use of the Bayley-III scale assessment tool (52). The abnormalities occurred mainly in the language domain during the first two years of life. The Z-score of the head circumference was significantly lower in the group with developmental delay, with the simultaneous presence of neurological abnormalities, which indicates a possible action of ZIKV infection in the developing brain (24).

Nielsen-Saines et al. (11) observed that, among the children evaluated by Bayley-III, 12% scored below two standard deviations (i.e., a score <70; a score of 100 ± 2 SD is the variation) in at least one functional domain; 28% of children scored between −1 and −2 SD in any domain (scores <85–70). Language function was most affected, with 35% of 146 children being below average. The authors described that neurodevelopmental outcomes were improved in female children, term babies, children with normal eye exames, and whose mothers were infected with ZIKV later in pregnancy. Mulkey et al. (47) found that infants with *in utero* ZIKV exposure without features of CZS were also at risk for abnormal neurodevelopment in the first 18 months of life.

### Eye Findings

Regarding ophthalmological findings, studies (12, 26, 67, 68, 73, 74, 76, 78–80) found an association between congenital infection due to presumed exposure to ZIKV and macular lesions, macular circumscribed chorio-retinal atrophy, focal-spotted retinal pigment epithelium, optic nerve pallor, early-onset strabismus, nystagmus, and low visual acuity. Also, ocular involvement (macular and eye fundus abnormalities) in babies with presumed congenital ZIKV infection was most frequently observed in babies with a smaller head circumference at the time of birth and whose mothers were infected in the first trimester of pregnancy (73).

### Hearing Deficits

Of the papers that assessed audiological function, the main findings were a statistically significant increase in latencies of waves I and III, compared to wave V, absence of otoacoustic emissions, and sensorineural hearing loss (37, 43). In most hearing loss cases associated with congenital infections, damage to the auditory system is due to cochlear involvement (81). Similar injuries are likely to be responsible for hearing loss in children with congenital ZIKV infection, although histological studies need to confirm this (39).

In a cross-sectional study, when evaluating 45 children with a mean age of 10 months, Leite et al. (41) found no association between exposure to ZIKV during pregnancy and audiological alterations. Similarly, when comparing children exposed and not exposed to ZIKV, Fandiño-Cárdenas et al. (30), in their cohort study of 66 exposed children did not observe hearing loss in the first two years of life.

In conclusion, hearing loss due to congenital ZIKV can be sensorineural, neural, conductive, isolated, or mixed. Therefore, a complete hearing assessment should be performed on all ZIKV-infected patients to rule out auditory neuropathy syndrome and sensorineural hearing loss (82).

### Cardiac Findings/Congenital Heart Disease

When analyzing the cardiovascular system of ZIKV-exposed children, Santana et al. (61) found echocardiographic abnormalities suggesting tropism of ZIKV to tissue beyond the central nervous system. Corroborating this finding, Orofino et al. (49) found a higher frequency of cardiac alterations in ZIKV-exposed babies than in the general population. However, none of these defects were severe. Therefore, the authors suggested that recommendations for performance of fetal echocardiograms in women with ZIKV infection during pregnancy and recommendations for postnatal infant echocardiogram should follow general infant population guidelines.

### Genito-Urinary Findings

All studies concerning genitourinary characteristics were performed in Brazil, two in the state of Rio de Janeiro and one in Pernambuco. Costa Monteiro et al. (22, 23) found that more than 90% of children with microcephaly in their series had neurogenic bladder, a health condition known to cause kidney damage when left untreated. On this theme, de Vasconcelos et al. (72) published a case series describing cryptorchidism in 3-year-old children with ZIKV-related microcephaly.

### Nutrition, Gastro-Intestinal Findings and Feeding Difficulties

Regarding the nutritional status of children exposed to ZIKV, nine papers described anthropometric changes such as low birth weight and growth restriction (20, 21, 28, 29, 33, 48, 50, 66).

In a cohort study, Soares et al. (63) found differences in arm and arm muscle circumference and fat-free mass in children from 1 to 3 months of age. Weight and length at 3 months of age were lower in ZIKV-exposed infants. Similarly, Carvalho-Sauer et al. (20) concluded that low birth weight in children with CZS was 4-fold greater as compared to children without CZS. Furthermore, prematurity and cesarean delivery were associated with low birth weight in exposed children. It should also be noted that most children with CZS were born to mothers of African heritage, single, and with less years of education, suggesting CZS disproportionately affected disenfranchised populations (28, 63).

Leal et al. (38) described a delay in the initial pharyngeal phase of swallowing. This combined with significant oral dysfunction, increases the risk of oral aspiration, predominantly with liquid foods. Also, Santana et al. (61) reported that four of 18 patients who had swallowing impairment were fed by gastrostomy.

In addition, Leal et al. (40) in a cross-sectional study nested in a cohort study, found a high prevalence of adenoid hypertrophy in children with Zika-related microcephaly, with consequent upper airway obstruction leading to chronic upper airway obstructive disorder, secretory otitis media and subsequent dysphagia (40). Abdominal imaging studies on the other hand showed no characteristic findings that were higher than those observed in the general population (55).

### Musculo-Skeletal Findings

Regarding orthopedic abnormalities, all seven papers described the presence of arthrogryposis in children with congenital zika, often present in both upper and lower extremities. A study by Aragão et al. (16) found that 75% of children with microcephaly and 100% of those with arthrogryposis had reduced thickness of the thoracic spinal cord. However, the latter group had evidence of narrowing of the entire spinal cord, with severely reduced spinal cord anterior roots. The authors concluded that it is crucial to consider Zika virus infection in the differential diagnosis of congenital diseases of the spinal cord and anterior nerve root if mother-infant pair have any risk factors for ZIKV antenatal exposure. This is especially relevant in mild cases where microcephaly is absent, and the only clinical manifestation is, for example, abnormal joints. On the other hand, health professionals should pay close attention when monitoring children from an epidemic area with mild or no clinical signs of spinal cord and anterior nerve root lesions, as they may have future problems with neuro-psychomotor development.

### Endocrinologic Findings

Regarding the endocrine system, the most prevalent and clinically relevant problems were pubertal dysfunctions, thyroid disease, growth faltering and obesity. These conditions require careful monitoring and highlight the need for endocrine evaluations in children with CZS, particularly those with microcephaly. Early diagnosis and referral to appropriate treatment in this situation may often be necessary (75).

### Need for Long Term Follow-Up

The repercussions of maternal infection during pregnancy on child development have been extensively described in the literature in regards to classic teratogenic pathogens responsible for TORCH syndromes (26). Fetal infection often triggers a systemic inflammatory response which may persist after birth, compounding further damage to the brain. This is one of the prevailing hypotheses on the pathogenesis of brain injury (26). Lesions associated with deep gray matter injury, vascular compromise and neural progenitor cell dysfunction have also been described (83–85).

Saad et al. (86) made the same recommendation in reviewing the most frequent clinical findings in children born to women with confirmed ZIKV infection during pregnancy. They described a broad spectrum of abnormalities resulting from an inflammatory reaction to the virus or a direct effect of the virus itself, causing damage to the CNS and neurological abnormalities which potentially manifest over time.

These published results describing developmental delay and other neuro-sensory deficits which may manifest later in life point to the need for continued monitoring of children with antenatal ZIKV exposure to assess risks of learning and behavioral disorders in the long term (85).

## Conclusion

In this systematic review, the most relevant findings were injuries to the infant central nervous system. CZS is a neurotropic disease with several associated abnormalities. Although the majority of published studies were from Brazil, there were no regional differences across the country and also in comparison to other countries in Latin America. Another important finding which the studies underscored is the later delay in development that may subsequently occur in an apparently normal infant at the time of birth. Finally, due to the vulnerability of women and children and the severe repercussions of ZIKV infection in pregnancy, studies should continue to monitor these children as they age. Broader descriptions of clinical findings are also important to further characterize the spectrum of disease in children. Prospective studies evaluating infants and children with antenatal ZIKV exposure may be able to describe the actual prevalence of adverse pregnancy, infant and childhood outcomes in this population. Prompt recognition of clinical abnormalities allows for implementation of early interventions which can improve later neurodevelopmental pathways in children born to mothers with gestational ZIKV infection.

## Data Availability Statement

The original contributions presented in the study are included in the article/supplementary material, further inquiries can be directed to the corresponding author/s.

## Author Contributions

YA, JM, and PGB did the manuscript search, reviewed all the literature and drafted the paper, and approved the final text. PB, KN-S, and MM formulated the research question, performed the analysis and draft of the paper, and approved the final text. All authors contributed to the article and approved the submitted version.

## Conflict of Interest

The authors declare that the research was conducted in the absence of any commercial or financial relationships that could be construed as a potential conflict of interest. The handling editor declared to have joint publications with the authors PB, KN-S, and MM.

## Publisher's Note

All claims expressed in this article are solely those of the authors and do not necessarily represent those of their affiliated organizations, or those of the publisher, the editors and the reviewers. Any product that may be evaluated in this article, or claim that may be made by its manufacturer, is not guaranteed or endorsed by the publisher.

## Abbreviations

ASD: autism spectrum disorder
BVS: biblioteca virtual em saude – (virtual health library)
CAPES: coordenação de aperfeiçoamento de pessoal de nível superior (thesis repository)
CNS: central nervous system
CZS: congenital zika syndrome
PRISMA: preferred reporting items for systematic reviews
SD: standard deviation
TORCH: toxoplasmosis, rubella, cytomegalovirus, herpes simplex
ZIKV: zika virus.
